# Chest radiograph reading and recording system: evaluation in frontline clinicians in Zambia

**DOI:** 10.1186/s12879-016-1460-z

**Published:** 2016-03-23

**Authors:** German Henostroza, Jennifer B. Harris, Nzali Kancheya, Venerandah Nhandu, Stable Besa, Robert Musopole, Annika Krüüner, Chisela Chileshe, Ian J. Dunn, Stewart E. Reid

**Affiliations:** Department of Medicine, Division of Infectious Diseases, University of Alabama at Birmingham, Birmingham, USA; Centre for Infectious Disease Research in Zambia, Lusaka, Zambia; Department of Epidemiology, University of Alabama at Birmingham, Birmingham, USA; Prisons Health Services, Ministry of Home Affairs, Lusaka, Zambia; Department of Radiology, University of British Columbia, Vancouver, Canada; Department of Medicine, Institute of Global Health and Infectious Diseases, University of North Carolina at Chapel Hill, Chapel Hill, USA

**Keywords:** chest radiograph, x-ray, Zambia, Chest Radiograph Reading and Recording System (CRRS)

## Abstract

**Background:**

In Zambia the vast majority of chest radiographs (CXR) are read by clinical officers who have limited training and varied interpretation experience, meaning lower inter-rater reliability and limiting the usefulness of CXR as a diagnostic tool. In 2010–11, the Zambian Prison Service and Ministry of Health established TB and HIV screening programs in six prisons; screening included digital radiography for all participants. Using front-line clinicians we evaluated sensitivity, specificity and inter-rater agreement for digital CXR interpretation using the Chest Radiograph Reading and Recording System (CRRS).

**Methods:**

Digital radiographs were selected from HIV-infected and uninfected inmates who participated in a TB and HIV screening program at two Zambian prisons. Two medical officers (MOs) and two clinical officers (COs) independently interpreted all CXRs. We calculated sensitivity and specificity of CXR interpretations compared to culture as the gold standard and evaluated inter-rater reliability using percent agreement and kappa coefficients.

**Results:**

571 CXRs were included in analyses. Sensitivity of the interpretation “any abnormality” ranged from 50–70 % depending on the reader and the patients’ HIV status. In general, MO’s had higher specificities than COs. Kappa coefficients for the ratings of “abnormalities consistent with TB” and “any abnormality” showed good agreement between MOs on HIV-uninfected CXRs and moderate agreement on HIV-infected CXRs whereas the COs demonstrated fair agreement in both categories, regardless of HIV status.

**Conclusions:**

Sensitivity, specificity and inter-rater agreement varied substantially between readers with different experience and training, however the medical officers who underwent formal CRRS training had more consistent interpretations.

**Electronic supplementary material:**

The online version of this article (doi:10.1186/s12879-016-1460-z) contains supplementary material, which is available to authorized users.

## Background

Despite global progress in tuberculosis (TB) prevention and control, TB remains a leading cause of morbidity and mortality in sub-Saharan Africa, especially among persons with HIV [1]. Accurate diagnosis is a major challenge with sputum-smear microscopy and chest radiography (CXR) still the primary diagnostic tools in many countries. Smear microscopy is less than 50 % sensitive in HIV-infected patients [2, 3], leaving many diagnoses reliant on CXR and clinical findings. In some settings, CXR is also used as a screening tool to identify TB suspects. Unfortunately CXR interpretation is complex and dependent on the skill of the reader and the quality of the x-ray.

Digital radiography has increased optimism for the use of CXRs as it offers consistent, better quality images and lower running costs than analog radiology [4, 5]. Other efforts to improve CXR accuracy focus on standardizing interpretation. An example is the Chest X-Ray Reading and Recording System (CRRS) form developed by the Lung Institute at the University of Cape Town [6, 7]. Experience with the CRRS to date has been reported primarily for sub-specialist providers, most of whom were certified as CRRS readers. [8–11] This requires a five-day training course [9], available only in South Africa, potentially limiting access for individuals from other countries with financial constraints.

In resource-limited settings (RLS) there is often a shortage of medical specialists resulting in CXR interpretation being performed by medical officers (MOs) and mid-level providers such as clinical officers (COs). With less training and experience in CXR interpretation than specialists, they likely have lower inter-rater reliability which further limits the usefulness of CXR as a diagnostic tool [12]. To date there is no data on the performance of CRRS in general practitioners and mid-level providers.

In Zambia the annual TB incidence is 427/100,000 and 64 % of TB patients are HIV-infected [1]. Only 29 % of HIV-infected pulmonary TB cases are smear-positive [13] and neither culture nor Xpert MTB/RIF are routinely available, so CXR plays an important role in TB diagnosis. However the vast majority of CXRs are read by clinical officers who have limited training and varied experience. We evaluated digital CXR interpretation using the CRRS form (Version 2007) in frontline clinicians in Zambia including two MOs and two COs.

## Methods

In 2010–11, the Zambian Prison Service and Ministry of Health established TB and HIV screening programs in six prisons with funding from the TB REACH initiative of the Stop TB Partnership and technical support from the Centre for Infectious Disease Research in Zambia (CIDRZ). The overall goal of this program was to develop capacity to ensure that TB and HIV screening were conducted for all inmates entering and residing in these facilities.

### TB and HIV screening protocol

Screening procedures have been described elsewhere [14]. Inmates were assessed for self-reported TB symptoms and other TB risk factors. Regardless of whether they had symptoms, all inmates submitted two sputa for florescence microscopy (FM), had a digital CXR taken, and underwent physical examination. The project CO made an initial TB diagnosis based on history, physical exam, CXR interpretation and FM smear results. In addition, inmates with an unknown HIV status were offered testing.

### Laboratory procedures

Two sputa per inmate underwent FM. One sputum per inmate was cultured using one of two algorithms: (a) both liquid (BD BACTEC™ MGIT™ 960 Mycobacteria Testing System) and solid (BD BBL™ Lowenstein-Jensen Medium) media or (b) two tubes of liquid media with the manual Mycobacteria Growth Indicator Tube system (BD BBL™ MGIT™ Mycobacterial Growth Indicator Tube). *M. tuberculosis complex* (MTBC) speciation and drug susceptibility was performed by Genotype MTBDR*plus* (Hain Life Sciences, Germany) line probe assay.

### Case definition

A TB case was defined as culture positive with species identified as MTBC.

### Chest X-ray selection

We selected a sample of CXRs as follows: (1) all patients with CXRs deemed abnormal by the project CO; (2) all persons with normal CXRs who were diagnosed with TB (based on smear results, clinical criteria, and/or culture confirmation); and (3) a random sample of inmates with normal CXRs and not diagnosed with TB. After evaluating the number of CXRs in categories (1) and (2), we decided to select 80 CXRs from HIV-positive and 80 from HIV-negative inmates in category (3) to strike a balance between feasibility to conduct all CXR readings and ensuring that there were an adequate number of normal CXRs in the sample. Our only exclusion criteria were current or recent TB treatment or having an unknown HIV status.

### Chest X-ray interpretation

Two Zambian MOs with over ten years of experience in diagnosing and treating tuberculosis patients and two COs with more than 5 years of experience were invited to participate in this evaluation. The MOs were Zambian graduates from the University of Zambia School of Medicine and the Zaparozhe Medical Institute, Ukraine; and the COs were local graduates of a 3-year program in clinical medicine, surgery and paediatrics. In addition, the two MOs attended the CRRS five-day training course in May 2010 in South Africa (using CRRS 2010 guidelines) where they were certified as “B-grade” readers. “B grade readers” are defined as those who take the course and pass the certification exam [15]. The COs did not attend the official CRRS training; instead they received a four-hour orientation provided by a non-CRRS trained radiologist who is a certified radiologist by the Royal College of Physicians of Canada and holds a faculty position within the Radiology Department at the University of British Columbia. The orientation consisted of a presentation and discussion of the CRRS form following the “Instructions for the use of the Chest Reading and Recording System” (using CRRS 2010 guidelines), followed by 20 h evaluating chest radiographs using the form.

The CRRS uses a simplified and systematic approach to CXR reading and interpretation with readers completing a form documenting CXR findings including the type of abnormality present (parenchymal, large/small opacifications, cavitation, pleural and central abnormalities) (Additional file 1). Readers are then required to make a final assessment whether the radiograph is “completely normal” and if not, whether the abnormalities found are “consistent with TB.” CXRs were read using the Rogan Delft View Pro-X (Version 4.0.8.4, Veenendal, NL) viewing software. Computer stations had monitors with a resolution of 1280 × 720 pixels. All readers were blinded to clinical data except for HIV status and did not have access to other readers’ reports.

### Data collection and analysis

Because we screened all inmates for TB, regardless of presenting characteristics, the vast majority were not diagnosed with TB and had CXRs that were classified as normal by the project CO. Thus we used disproportionate stratified sampling to maximize the number of abnormal CXRs included in this study. We selected all CXRs deemed “abnormal” by the study CO as well as all CXRs from inmates diagnosed with TB. We selected a subset of CXRs from patients who were not diagnosed with TB and were deemed to have “normal” CXRs by the study CO.

Data were collected using CRRS forms configured into an electronic format using MS Access (Microsoft) and Visual Basic (Microsoft). Readers entered data directly into the electronic record. The system had features to minimize data entry errors including consistency checks and automatic skips. All data were exported into SAS 9.3 (Cary, North Carolina, USA) for subsequent cleaning and analysis.

We calculated sensitivity and specificity of the CXR interpretations “any abnormality” and “abnormalities consistent with TB” for each reader using TB culture as the gold standard. We assessed inter-rater reliability with percent agreement and kappa coefficients between the two CRRS-certified MOs (Reader 1 & Reader 2) and the two CRRS-oriented COs (Reader 3 & Reader 4). Percent agreement and kappa coefficients were calculated for 8 major abnormality classifications on the CRRS system: parenchymal abnormalities, large opacifications, small opacifications, cavitation, pleural abnormalities, central abnormalities, any abnormality and abnormalities consistent with TB. Ninety-five percent confidence intervals were calculated for all measures. Kappa coefficients were interpreted as follows: ≤0.2 was considered poor agreement; 0.21–0.40 was fair; 0.41–0.60 was moderate; 0.61–0.80 was good; and >0.80 was very good. Performance measures were compared between clinician groups (MOs and COs) for obvious trends. Because each group had only two readers that were selected for convenience, we did not summarize measures within clinician groups or conduct statistical tests to compare groups.

#### Ethics statement

The protocol was approved by the Biomedical Research Ethics Committee of the University of Zambia (001–03–11), the Zambian Ministry of Health and the Institutional Review Boards of the University of Alabama at Birmingham (F101014011) and the University of North Carolina at Chapel Hill, United States of America.

A waiver of informed consent and documentation was approved by both the above named ethics committees. This was a retrospective analysis of de-identified electronic data collected under a previously approved protocol and stored using a unique identifying number, meaning it was not feasible to trace all participants screened within the program.

## Results

Between January and July 2011, 3405 inmates without a current or recent history of TB were screened for TB and HIV. 3160 agreed to HIV testing or had a known prior status. From 711 HIV-positive inmates, 235 CXRs were selected as follows: 137/137 CXRs deemed abnormal by the project CO; 18/18 normal CXRs from patients who were diagnosed with TB based on smear, culture, and/or clinical criteria; and 80/556 normal CXRs randomly selected from patients not diagnosed with TB. From 2449 HIV-negative inmates, 339 CXRs were selected as follows: 236/236 CXRs deemed abnormal by the project CO; 23/23 normal CXRs from patients who were diagnosed with TB based on smear, culture, or clinical criteria, and 80/2190 normal CXRs randomly selected from patients not diagnosed with TB. Of the 574 images selected, three could not be interpreted due to file corruption.

### Patients and case description

Of 571 patients included in analyses, 233 (41 %) were HIV-infected. The inmates’ mean age was 38.6 years, 97.4 % were male, and 73.6 % presented with at least one TB symptom. One fourth of them (25.2 %) had a prior history of TB and 503 (88.1 %) had a valid culture result. Of these, 74 (14.7 %) had culture-confirmed TB; 30/200 (15 %) among HIV-infected and 44/303 (14.5 %) among HIV-uninfected (Table 1).

**Table 1:** Cohort characteristics

Characteristic	HIV positive participants	HIV negative participants	All participants
	*n* = 233	*n* = 338	*n* = 571
Male sex	220 (94.4 %)	336 (99.4 %)	556 (97.4 %)
Age, mean (SD)	38.1 (8.4)	39.0 (12.9)	38.6 (11.3)
Prior history of TB^a^	83 (35.6 %)	61 (18.0 %)	144 (25.2 %)
Culture-confirmed TB^b^	30/200 (15.0 %)	44/303 (14.5 %)	74/503 (14.7 %)
Smear positive TB	9 (3.9 %)	14 (4.1 %)	23 (4.0 %)
Any TB-related symptoms^a^	179 (76.8 %)	241 (71.3 %)	420 (73.6 %)

^a^Self-reported cough, fever, weight loss, night sweats, difficulty breathing, chest pain, loss of appetite, or swelling (lymphadenopathy)

^b^68 patients excluded due to missing/contaminated cultures

### Chest X ray sensitivity and specificity compared to culture

Sixty-eight participants (33 HIV-positive and 35 HIV-negative) had missing or contaminated cultures and were excluded from sensitivity and specificity analyses. We first assessed sensitivity and specificity of the classification “abnormalities consistent with TB”. Compared to culture, the CRRS-certified MOs’ readings had sensitivities of 57 and 50 % and specificities of 61 and 60 % in CXRs from HIV-infected inmates. For CXRs from HIV-uninfected inmates, the MOs’ sensitivities were 61 and 55 % with specificities of 70 and 55 %. The CRRS-oriented COs had sensitivities 67 and 53 % with specificities of 42 and 37 % in HIV-infected inmates. Among HIV-uninfected patients, sensitivities for COs were 68 and 61 % with specificities of 38 and 37 %. When we broadened the CXR classification to “any abnormalities,” point estimates for sensitivity increased slightly for two of the four readers, but did not change for the other two (Table 2).

**Table 2:** Sensitivity & Specificity compared to culture, stratified by HIV status

	HIV Positive				HIV Negative			
	*n* = 30 culture-confirmed TB; 170 no TB^a^				*n* = 44 culture-confirmed TB; 259 no TB			
	Abnormalities consistent with TB		Any abnormality		Abnormalities consistent with TB		Any abnormality	
	Sensitivity	Specificity	Sensitivity	Specificity	Sensitivity	Specificity	Sensitivity	Specificity
CRRS-certified medical officers								
Reader 1	0.57	0.61	0.63	0.50	0.61	0.59	0.70	0.47
	(0.37–0.75)	(0.53–0.68)	(0.44–0.80)	(0.42–0.58)	(0.45–0.76)	(0.53–0.65)	(0.54–0.83)	(0.41–0.54)
Reader 2	0.50	0.60	0.50	0.59	0.55	0.61	0.55	0.60
	(0.31–0.69)	(0.52–0.67)	(0.31–0.69)	(0.51–0.66)	(0.39–0.70)	(0.55–0.67)	(0.39–0.70)	(0.54–0.66)
CRRS-oriented clinical officers								
Reader 3	0.67	0.42	0.77	0.38	0.68	0.38	0.70	0.33
	(0.47–0.83)	(0.34–0.50)	(0.58–0.90)	(0.31–0.46)	(0.52–0.81)	(0.32–0.44)	(0.55–0.83)	(0.27–0.39)
Reader 4	0.53	0.37	0.53	0.35	0.61	0.37	0.61	0.33
	(0.34–0.72)	(0.30–0.45)	(0.34–0.72)	(0.28–0.43)	(0.45–0.76)	(0.32–0.44)	(0.45–0.76)	(0.27–0.39)

^a^33 HIV–positive and 35 HIV–negative participants excluded due to missing or contaminated cultures

With both classifications (“abnormalities consistent with TB” and “any abnormalities”), three of the four readers had slightly higher sensitivities with CXRs from HIV-negative persons than with CXRs from HIV-positive persons (Table 2), however all differences were small and confidence intervals overlapped substantially. Comparing the CRRS-certified MOs to the CRRS-oriented COs, the only consistent trend was higher *specificities* for the MOs (Table 2).

### Inter rater reliability

#### Percent agreement

Percent agreement and kappa statistics are shown in Table 3. Percent agreement between CRRS-certified MOs on identification of specific abnormalities ranged from 73 to 87 % for HIV-infected patients and 76 to 96 % for HIV-uninfected patients. For CRRS-oriented COs, percent agreements ranged from 65 to 93 % in HIV-infected patients and 69 to 84 % in HIV-uninfected patients.

**Table 3:** Inter–rater reliability

Agreement Index	Parenchymal abnormalities	Large opacifications	Small opacifications	Cavitation	Pleural abnormalities	Central abnormalities	Any abnormality	Abnormalities consistent with TB
CRRS–certified medical officers								
HIV Positive patients (*N* = 231)^a^								
Both readers agree abnormality present	38	8	24	2	32	17	78	69
Only Reader 1 says abnormality present	56	38	36	30	13	21	41	28
Only Reader 2 says abnormality present	7	5	10	0	16	17	22	29
Both readers agree abnormality not present	130	180	161	199	170	176	90	105
Percent Agreement (95 % CI)	0.73	0.81	0.80	0.87	0.87	0.84	0.73	0.75
	(0.67–0.78)	(0.76–0.86)	(0.75–0.85)	(0.83–0.91)	(0.83–0.92)	(0.79–0.88)	(0.67–0.78)	(0.70–0.81)
Kappa (95 % CI)	0.38	0.20	0.40	0.10	0.61	0.38	0.46	0.49
	(0.27–0.50)	(0.06–0.35)	(0.26–0.53)	(–0.03–0.24)	(0.48–0.74)	(0.22–0.54)	(0.34–0.57)	(0.38–0.61)
Strength of agreement (based on kappa)	Fair	Poor	Fair	Poor	Good	Fair	Moderate	Moderate
HIV Negative patients (*N* = 335)^a^								
Both readers agree abnormality present	58	10	41	6	60	18	136	122
Only Reader 1 says abnormality present	100	74	64	39	24	32	55	31
Only Reader 2 says abnormality present	5	1	12	0	17	15	9	19
Both readers agree abnormality not present	172	250	218	290	234	270	135	163
Percent Agreement (95 % CI)	0.96	0.78	0.77	0.88	0.88	0.86	0.76	0.85
	(0.64–0.74)	(0.73–0.82)	(0.73–0.82)	(0.85–0.92)	(0.84–0.91)	(0.82–0.90)	(0.72–0.81)	(0.81–0.89)
Kappa (95 % CI)	0.35	0.16	0.39	0.21	0.66	0.36	0.62	0.70
	(0.27–0.43)	(0.07–0.26)	(0.29–0.50)	(0.07–0.35)	(0.57–0.76)	(0.21–0.50)	(0.54–0.70)	(0.62–0.77)
Strength of agreement (based on kappa)	Fair	Poor	Fair	Fair	Good	Fair	Good	Good
CRRS-oriented clinical officers								
HIV Positive patients (*N* = 231)^a^								
Both readers agree abnormality present	46	33	5	1	55	14	107	102
Only Reader 3 says abnormality present	33	28	22	5	16	41	40	35
Only Reader 4 says abnormality present	44	37	11	11	63	21	41	43
Both readers agree abnormality not present	108	133	193	214	97	155	43	51
Percent Agreement	0.67	0.72	0.86	0.93	0.66	0.73	0.65	0.66
	(0.61–0.73)	(0.66–0.78)	(0.81–0.90)	(0.90–0.96)	(0.60–0.72)	(0.67–0.79)	(0.59–0.71)	(0.60–0.72)
Kappa (95 % CI)	0.28	0.31	0.16	0.08	0.32	0.15	0.24	0.29
	(0.16–0.41)	(0.18–0.44)	(–0.02–0.34)	(–0.12–0.28)	(0.21–0.43)	(0.01–0.29)	(0.11–0.37)	(0.17–0.42)
Strength of agreement (based on kappa)	Fair	Fair	Poor	Poor	Fair	Poor	Fair	Fair
HIV Negative patients (*N* = 335)^a^								
Both readers agree abnormality present	88	58	9	1	107	36	177	165
Only Reader 3 says abnormality present	42	38	43	10	18	70	52	51
Only Reader 4 says abnormality present	36	48	11	15	86	22	48	48
Both readers agree abnormality not present	169	191	272	309	124	207	58	71
Percent Agreement (95 % CI)	0.77	0.74	0.84	0.92	0.69	0.73	0.70	0.70
	(0.72–0.81)	(0.70–0.79)	(0.80–0.88)	(0.90–0.95)	(0.64–0.74)	(0.68–0.77)	(0.65–0.75)	(0.66–0.75)
Kappa (95 % CI)	0.51	0.39	0.18	0.04	0.40	0.28	0.32	0.36
	(0.41–0.60)	(0.28–0.50)	(0.05–0.31)	(–0.10–0.18)	(0.31–0.49)	(0.17–0.38)	(.21–0.42)	(0.25–0.46)
Strength of agreement (based on kappa)	Moderate	Fair	Poor	Poor	Fair	Fair	Fair	Fair

^a^2 CXRs among HIV-positive and 3 among HIV-negative patients were deemed ‘Unreadable’ by one or more reader and excluded from analyses

#### Kappa coefficient

The MOs’ kappa coefficients for “abnormalities consistent with TB,” were 0.49 for HIV-positive and 0.70 for HIV-negative CXRs. Kappas for “any abnormality” were 0.46 for HIV-positive and 0.62 for HIV-negative CXRs. Kappa coefficients for specific chest abnormalities (cavities, opacifications and pleural, central or parenchymal abnormalities) ranged from 0.35 to 0.61 among HIV-infected and from 0.29 to 0.66 among HIV-uninfected inmates.

The COs had kappa coefficients for “abnormalities consistent with TB” of 0.29 with HIV-infected and 0.36 with HIV-uninfected CXRs. For “any abnormality” the COs had kappas of 0.24 in HIV-positive and 0.32 in HIV-negative CXRs. Kappas for specific chest abnormalities ranged from 0.08 to 0.32 among HIV-infected and from 0.04 to 0.51 among HIV-uninfected.

## Discussion

The value of CXR for TB screening and diagnosis has shown wide variability in performance across different settings and patient populations [3, 9, 16, 17]. We evaluated the performance of digital radiography when interpreted by front-line clinicians using CRRS forms in Zambia. We provided COs with a local orientation to the CRRS form to assess their performance with the underlying rationale that the five-day CRRS training course in South Africa is not easily accessible to COs in Zambia who perform much of the CXR interpretation for TB diagnosis.

Despite using digital radiographs, the sensitivity of the interpretation “any abnormality” ranged from only 50–70 % depending on the reader and the patients’ HIV status. Thus if CXR was used as the sole TB *screening* tool in this cohort, 30 to 50 % of the culture-confirmed TB cases would have been missed. Even more cases may have been missed if the rating “abnormalities consistent with TB” was used, as sensitivities were slightly lower for two of the four readers. The use of CXR abnormalities as *diagnostic* criteria may result in over-diagnosis of cases since specificities for the classification “abnormalities consistent with TB” ranged from 37–61 %. When comparing the CRRS-trained MOs to the CRRS-oriented COs, the MOs consistently had higher specificities. In contrast, there were no consistent trends seen with sensitivities. Furthermore, sensitivities were not strongly influenced by HIV status.

We looked at inter-rater reliability using kappa statistics. For the ratings of “abnormalities consistent with TB” and “any abnormality” the MO’s had “good” agreement for HIV-uninfected CXRs but only “moderate” agreement for HIV-infected CXRs. This is consistent with literature showing that HIV-infected TB patients present with broad array of atypical radiological abnormalities [18–21]. When looking at specific types of abnormalities, the agreement between MOs was very similar for HIV-infected and HIV-uninfected patients, but their agreement was “good” only for pleural abnormalities; the rest were in the “poor” or “fair” ranges. This suggests that MOs had better agreement on overall interpretation of CXRs than on specific abnormalities.

The CRRS-oriented COs demonstrated “poor” or “fair” agreement for almost all categories. Unlike the MOs, they did not have better agreement on the overall assessment categories than they did for the specific abnormalities. Looking at specific abnormalities, they achieved “moderate” agreement for parenchymal abnormalities in HIV-negative patients and “fair” agreement for large opacities and pleural abnormalities. This agreement in identifying gross, more easily observable radiographic abnormalities might be expected given their level of training. Some of the lowest kappas for both MOs and COs were observed with cavities, which is a concern given the high correlation of this abnormality with pulmonary TB. Other studies have similar findings, even with expert readers [9]. Readers were not blinded for HIV status to reflect actual case scenario when evaluating TB suspects. Due to the Zambian opt out approach to HIV testing most TB suspects will have an HIV test result that can be accessed by the radiologist.

The explanation for differences in performance of sensitivity and specificity between MOs and COs is likely multifactorial but likely most related to the level of health care worker training. With only a three-year training program, the COs had less clinical instruction, training, and mentorship. In addition, our study COs did not complete the formal CRRS training. Our evaluation was based on an active case finding intervention in a high risk population. Therefore, other pathologies could be responsible for abnormal radiological findings in conjunction with clinicians with a high index of suspicion for TB.

The low kappa statistics among COs suggests that a short orientation to the CRRS form is not sufficient to develop acceptable CXR interpretation skills for people with their level of training. Since the formal CRRS training is inaccessible to most frontline clinicians outside of South Africa, alternatives could include a “Trainer of Trainers” curriculum such that CRRS-certified “trainers” could return to their respective sites to train and mentor others. Ongoing mentoring to address skills deficits of frontline providers might also be accomplished by an e-learning curriculum that provides reminders/refreshers using text and images demonstrating variations of radiographic abnormalities [22]. When designing x-ray training packages for COs, special attention should be placed on x-ray interpretation in HIV-infected patients and recognition of cavities, given its high correlation with pulmonary tuberculosis [23]. A more simple classification for CXR interpretation could improve sensitivity and inter-rater reliability between clinical officers [24].

These results highlight some of the challenges of using CXR as a primary screening and/or diagnostic tool for non-radiologists. Sensitivity, specificity and inter-rater agreement varied substantially based on reader experience and training. The sensitivity and specificity of CXR, as well as the training of the health care providers who will be interpreting CXRs, should be carefully considered when implementing chest radiography in screening and diagnostic algorithms. In addition, these findings suggest that prison environments warrant a high index of suspicion for TB even among inmates with normal CXRs since the sensitivity of CXR was fairly low, regardless of who was reading the CXR.

### Strengths and limitations

This evaluation had several strengths: the electronic CRRS form limited data transcription errors and the high quality of digital CXRs should have minimized inconsistencies due to poor quality radiographs. Other strengths are the inclusion of both HIV-infected and uninfected, symptomatic and asymptomatic patients who had culture results to serve as a gold standard for TB diagnostic status. This provided a diverse study population in which to evaluate inter-observer agreement and diagnostic performance.

We also had a few limitations. First of all, we had only two MOs and two COs so it is difficult to make generalizations about classes of frontline providers, especially since the MOs had received CRRS training but the COs had not. Another limitation is that our radiologist was not CRRS trained, this could have affected the correct interpretation of the CRRS form as was intended to be used. In addition, we only cultured one sputum per person which could have resulted in a few patients who truly had TB being classified as “TB negative.” If this happened, it could have resulted in slightly lower specificities for CXR interpretation. However, all persons were cultured with both liquid and solid culture and we do not believe this would have had a substantial effect on results. Because we screened all inmates, regardless of presenting characteristics, many TB patients were probably caught at an early stage of disease. As such, their CXRs may not be typical of patients with more advanced TB disease. Also due to our screening setting, we elected to use a non-random sample of CXRs to ensure that the selection included culture-confirmed cases (for sensitivity assessment) as well as abnormal CXRs for assessment of agreement. However the COs and MOs were blinded to the CXR selection process, thus this should not have subjectively affected their assessments. Finally, Kappa statistics are affected by the prevalence of the assessed condition [25] and thus should be interpreted with caution. A study in Kenya found lower kappas when assessing CXRs from persons without TB than CXRs from TB patients [24]. This suggests that kappas in our study may have been even lower had there been a higher proportion of CXRs from persons without TB.

## Conclusions

The WHO’s 2013 guidelines for systematic TB screening recommend use of chest radiography for TB screening as it is more sensitive than symptom-based algorithms [26]. Our study suggests that this approach may not be highly sensitive in some settings and may be limited by poor consistency in CXR interpretation among frontline providers. The CRRS system creates a structure for CXR interpretation which should be helpful for novice or less experienced readers, however we found that the CRRS form alone is not a substitute for (a) formal training/experience in identifying specific CXR abnormalities (b) knowledge/experience in deciding which abnormalities are likely to be caused by TB. However in settings with few radiologists, a tool such as CRRS might represent a viable option if combined with onsite trainers, mentoring and constant feedback.

## Additional file

Additional file 1: Chest Radiograph Reading and Recording System. (PDF 247 kb)

## Abbreviations

CIDRZ: Centre for Infectious Disease Research in Zambia
COs: Clinical officers
CRRS: Chest Radiograph Reading and Recording System
CXR: Chest radiograph
FM: Florescence microscopy
MOs: Medical officers
MTBC: *M. tuberculosis complex*
RLS: Resource-limited settings
TB: Tuberculosis
