# Supplementary material for: Chest radiograph reading and recording system: evaluation in frontline clinicians in Zambia
Source: BMC Infect Dis. 2016 Mar 23;16:136. doi: 10.1186/s12879-016-1460-z (PMC4804604; doi:10.1186/s12879-016-1460-z)
Supplement: Additional file 1: — Chest Radiograph Reading and Recording System. (PDF 247 kb) [file 12879_2016_1460_MOESM1_ESM.pdf]

CHEST RADIOGRAPH READING AND RECORDING SYSTEM

Use a dark pen   Cross boxes that apply   Do not mark anywhere outside the boxes   Use white stickers to make corrections

Program ID: ☐☐-☐☐☐☐-☐☐   Sex: ☐ Male ☐ Female

Patient Initials: ____________   HIV Results: ________________

Patient Date Of Birth: ☐☐ DD ☐☐ MM ☐☐☐☐ YYYY

Full Name of Person Completing the Form

Reader: ______________________________

Reading Date: ☐☐ ☐☐ ☐☐☐☐

Film Quality ☐ Optimal ☐ Suboptimal ☐ Unreadable

Comments ☐ Too dark/too light ☐ Poor position ☐ Artifact Other, specify: ________________

**Parenchymal abnormalities** ☐ Y ☐ N					
	**Type**	**Size**	**Extent**	**Zones** (R / L)	**Calcification**
**1A Large Opacities(>1cm)** ☐ Y ☐ N	☐ Round<br>☐ Irregular	☐ 1 - 5 cm<br>☐ > 5 cm<br>☐ > upper lobe	☐ Single<br>☐ Few<br>☐ Many	U ☐ ☐<br>M ☐ ☐<br>L ☐ ☐	☐ Yes<br>☐ No
	**Type**	**Size**	**Profusion**	**Zones** (R / L)	**Calcification**
**1B Small opacities (<1cm)** ☐ Y ☐ N	☐ Round<br>☐ Irregular	☐ <1.5 mm<br>☐ 1.5 - 3.5mm<br>☐ 3.5 - 10 mm	☐ 1 +<br>☐ 2 +<br>☐ 3 +	U ☐ ☐<br>M ☐ ☐<br>L ☐ ☐	☐ Yes<br>☐ No
		**Maximum size**	**Extent**	**Zones** (R / L)	
**1C Cavities** ☐ Y ☐ N		☐ 1 - 5 cm<br>☐ > 5 cm<br>☐ > upper lobe	☐ Single<br>☐ Few<br>☐ Many	U ☐ ☐<br>M ☐ ☐<br>L ☐ ☐	

**Pleural abnormalities** ☐ Y ☐ N	**Chest side**		**Extent of lateral chest wall**		
2A Calcification/Plaque ☐ Y ☐ N	☐ R	☐ L	☐ <1/4	☐ 1/4 - 1/2	☐ >1/2
2B Pleural fluid/Fibrosis ☐ Y ☐ N	☐ R	☐ L	☐ <1/4	☐ 1/4 - 1/2	☐ >1/2
2C Apical cap ☐ Y ☐ N	☐ R	☐ L			

**Central abnormalities** ☐ Y ☐ N					
**3A Tracheal deviation** ☐ Y ☐ N		☐ R	☐ L		
**3B Mediastinal shift** ☐ Y ☐ N		☐ R	☐ L		
**3C Hilar elevation** ☐ Y ☐ N		☐ R	☐ L		
**3D Lymphadenopathy** ☐ Y ☐ N	**Hilar**	☐ R	☐ L	**Mediastinal** ☐ R	☐ L

Other abnormalities ☐ Y ☐ N

Surgical
- ☐ Bullets/Artifact/Foreign body
- ☐ Suspected lung resection
- ☐ Sternotomy wire/clips

Cardiac
- ☐ Enlarged
- ☐ Any Abnormality

Skeletal
- ☐ Rib fracture or abnormality
- ☐ Spinal abnormality

Lung
- ☐ Bullae
- ☐ Bronchiectasis
- ☐ Suspected cancer

Lung
- ☐ Hyperinflation
- ☐ Pneumothorax
- ☐ Mycetoma
- ☐ Volume loss

☐ Y ☐ N Radiograph completely normal

☐ Y ☐ N Abnormalities consistent with TB

If No, specify ____________________
